# Function-preserving gastrectomy based on the sentinel node concept prevents osteosarcopenia in patients with gastric cancer

**DOI:** 10.1007/s10120-025-01617-7

**Published:** 2025-04-27

**Authors:** Yuki Hirase, Takaaki Arigami, Daisuke Matsushita, Masataka Shimonosono, Yoshikazu Uenosono, Shigehiro Yanagita, Yusuke Tsuruda, Ken Sasaki, Kenji Baba, Yota Kawasaki, Takao Ohtsuka

**Affiliations:** Department of Digestive Surgery, Kagoshima University Graduate School of Medical and Dental Sciences, 8-35-1 Sakuragaoka, Kagoshima, 890-8520 Japan

**Keywords:** Gastric cancer, Sentinel node navigation surgery, Osteosarcopenia

## Abstract

**Background:**

Gastric cancer remains a significant global challenge, with conventional surgery for early gastric cancer often leading to post-gastrectomy complications. Sentinel node navigation surgery is being developed to preserve quality of life without compromising radicality. Although osteosarcopenia is linked to gastrointestinal cancers and prognosis, its impact on bone and muscle mass after function-preserving surgery for gastric cancer remains underexplored.

**Methods:**

We analyzed the data of patients diagnosed with early gastric cancer and not eligible for endoscopic treatments, who underwent either distal gastrectomy or sentinel node navigation surgery at our hospital between 2010 and 2020. Skeletal muscle index and bone mineral density were measured preoperatively and 1, 3, and 5 years, postoperatively; rates of changes in these measures were assessed.

**Results:**

Among the 63 patients included, 42 (67%) underwent conventional surgery, and 21 (33%) underwent function-preserving gastrectomy using the sentinel node technique. No significant difference in postoperative survival rates was observed between the two groups (*P* = 0.97). The rate of change in the skeletal muscle index and bone mineral density decreased in both groups from 1 to 3 years postoperatively. At 5 years postoperatively, the sentinel node navigation surgery group showed an increase in skeletal muscle index and bone mineral density change rates, the difference observed between the two groups was significant (*P* < 0.05).

**Conclusion:**

Sentinel node navigation surgery for early gastric cancer may help prevent decreases in bone and muscle mass. This suggests that its use has a potential role in preventing osteosarcopenia.

## Introduction

Gastric cancer is the fifth most common malignancy worldwide and the third leading cause of cancer-related mortality [1]. According to Japanese treatment guidelines for gastric cancer, early gastric cancer (EGC) that is not eligible for endoscopic treatment is typically treated with conventional gastrectomy with lymphadenectomy [2]. However, because lymph node metastasis occurs in only 15–20% of patients with EGC, many patients without metastasis undergo unnecessary extensive lymphadenectomy and gastrectomy, leading to postoperative complications such as reduced food intake, body weight loss, dumping syndrome, and other gastrointestinal symptoms [3]. Therefore, accurate evaluation of lymph node metastasis is crucial in determining appropriate treatments for EGC [4].

The sentinel node (SN) is defined as the first regional lymph node that receives direct lymphatic drainage from the primary tumor and it is the most likely site of early metastasis [5]. If no metastasis is detected in the sentinel node, other regional lymph nodes are unlikely to harbor cancer, allowing for lymphadenectomy to be replaced by function-preserving surgery instead. Morton et al. first proposed the sentinel node concept in 1992 [6]. Sentinel node navigation surgery (SNNS) has been validated in breast cancer and melanoma [6, 7]: the technique may offer means by which quality of life can be maintained after surgery for gastric cancer without compromising radicality or safety [8, 9].

Recently, the combined presence of sarcopenia and osteopenia, referred to as osteosarcopenia, has gained attention [10]. Sarcopenia is characterized by decreased skeletal muscle mass and strength, whereas osteopenia indicates decreased bone density. Both conditions are associated with poor prognosis in gastric cancer [11, 12]. Previously, we reported that osteosarcopenia represents an additional prognostic factor in gastric cancer [13]. However, the clinical impact of function-preserving surgery based on the concept of a sentinel node on changes in bone and muscle mass in EGC remains insufficiently explored.

Therefore, this study evaluated the effects of SNNS and conventional gastrectomy on changes in bone mineral density (BMD) and skeletal muscle mass in patients with EGC. We conducted this evaluation to determine whether using a specific surgical approach can prevent postoperative osteosarcopenia.

## Methods

### Patients

A retrospective analysis was conducted involving patients diagnosed with gastric cancer at stages cT1 or cN0. These patients were ineligible for endoscopic treatment and underwent surgical resection at Kagoshima University Hospital between January 2010 and December 2020. The patients were excluded if they had not undergone postoperative computed tomography (CT) examinations at our institution. All patients underwent blood tests, esophagogastroduodenoscopy, and CT prior to gastrectomy.

Informed consent was obtained using an opt-out method. This retrospective study was approved by the Ethics Committee of Kagoshima University (approval number 240131).

### Surgical procedures

The surgical procedures performed included laparoscopic local resection (LLR) based on SNNS or laparoscopic distal gastrectomy (LDG). On the day before surgery, 3 mCi (2 mL) of technetium-99 m tin colloid was endoscopically injected into the submucosal layer at four sites (0.5 mL at each site) surrounding the tumor. Similarly, indocyanine green was injected preoperatively. During surgery, radioisotope uptake in each lymph node was measured using the Navigator GPS (Tyco Healthcare Japan Inc., Tokyo, Japan), and indocyanine green fluorescence was detected using an infrared imaging system (Olympus Corporation, Tokyo, Japan). The sentinel node was identified and sampled. The absence of lymph node metastasis was confirmed with an intraoperative rapid pathological examination before performing LLR. If lymph node metastasis was detected during the intraoperative rapid pathological examination, LDG with D2 lymphadenectomy was performed.

### Measurement of skeletal muscle index and BMD

Preoperative and 1-, 3-, and 5-year postoperative CT images were analyzed using the Volume Analyzer SYNAPSE VINCENT image analysis system (Fujifilm Medical, Tokyo, Japan) to evaluate body composition indices.

The temporal changes in skeletal muscle mass were compared using the skeletal muscle index (SMI) measured at the level of the L3 vertebra [14]. The tissue Hounsfield-unit threshold for skeletal muscle ranges from − 29 to 150 Hounsfield units [14]. The SMI (measured in cm^2^/m^2^) was calculated by dividing the skeletal muscle area (cm^2^) by the square of the patient’s height (m^2^) [15]. The rate of change in the SMI was calculated by subtracting the previous year’s SMI from the target SMI and dividing that value by the previous year’s SMI.

Temporal changes in BMD were compared by examining the mean pixel density within an elliptical region of interest at the level of the Th11 vertebra [15]. Similar to the rate of change in the SMI, that of BMD was calculated by subtracting the previous year’s BMD from the target BMD and dividing that value by the previous year’s BMD.

**Fig. 1:**
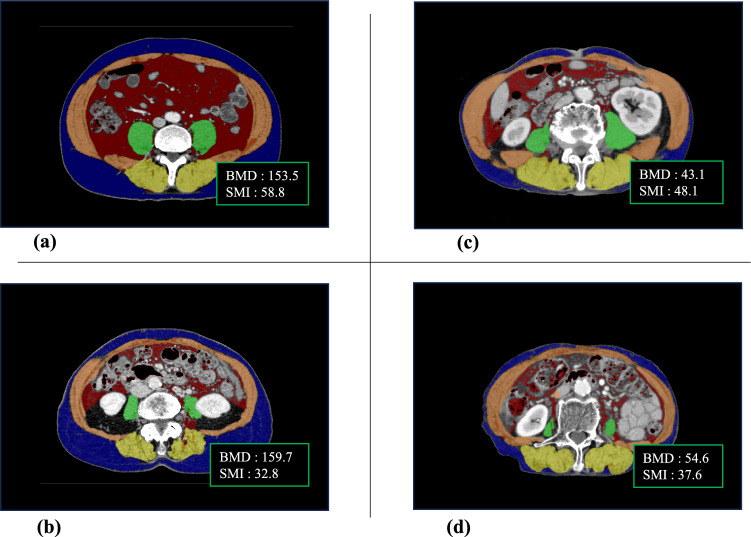
Representative computed tomography images showing body composition ranges. Ranges of body composition include within normal (**a**), with sarcopenia—decreased skeletal muscle index (**b**), with osteopenia—decreased bone mineral density (**c**), and with osteosarcopenia—decreased skeletal muscle index and bone mineral density (**d**).

To enhance accuracy, both the SMI and BMD were measured at two points on the CT images and the average value was calculated. Figure 1 shows the representative CT images for a normal SMI and BMD range, sarcopenia, osteopenia, and osteosarcopenia.

### Blood markers for nutrition and systemic inflammatory response

In this study, the neutrophil-to-lymphocyte ratio (NLR), prognostic nutritional index (PNI), C-reactive protein to albumin ratio (CAR), and albumin levels were used as blood markers for nutritional and systemic inflammatory responses. The blood samples were collected before surgery. Neutrophils, lymphocytes, and platelets were counted using an XN-20 automated hematology analyzer (Sysmex Corporation, Kobe, Japan). C-reactive protein and albumin levels were measured using a JCA-ZS050 automated analyzer (JEOL Ltd., Tokyo, Japan).

The NLR was calculated by dividing the number of neutrophils by the number of lymphocytes. PNI was determined as serum albumin (g/L) × 10 + 0.005 × total lymphocyte count (per mm^3^). CAR was calculated by dividing the C-reactive protein level by the albumin level. The cutoff values for the NLR, PNI, CAR, and albumin levels in the univariate and multivariate analyses were set using the median values.

### Statistical analyses

The relationship between surgical procedures and clinicopathological factors was evaluated using the chi square, Fisher’s exact, and Wilcoxon rank-sum tests. Overall survival was defined as the period from surgery to death or the final follow-up. Kaplan–Meier survival curves were constructed and differences in prognosis were determined using the log-rank test. All statistical analyses were performed using JMP software (SAS Institute Inc., Cary, NC, USA). Significance was set at *P* < 0.05.

## Results

### Relationship between surgical procedures and preoperative clinicopathological factors

Of the 120 eligible participants, 57 were excluded because they had not undergone postoperative CT examinations at our institution. Consequently, 63 patients (36 male, 27 female) were included. The preoperative clinicopathological characteristics of the 63 patients, categorized into the LLR and LDG groups, are summarized in Table 1. The median age in the LLR and LDG groups was 68 and 67.5 years, respectively (*P* = 0.84). No significant differences were observed in median body weight or body mass index (BMI) between the two groups (*P* = 0.47 and *P* = 0.79, respectively). Regarding tumor location, tumors in the upper stomach were significantly more frequent in the LLR group, while those in the lower stomach were significantly more frequent in the LDG group (*P* < 0.01). The distribution of normal, osteopenia, sarcopenia, and osteosarcopenia showed no significant differences between the two groups (*P* = 0.50). Additionally, no significant differences were noted in the median preoperative values of NLR, PNI, CAR, or albumin between the two groups (*P* = 0.27, *P* = 0.30, *P* = 0.88, *P* = 0.28, *P* = 0.31, and *P* = 0.14, respectively).

**Table 1:** Relationship between surgical procedures and preoperative clinicopathological characteristics (*N* = 63)

Characteristic	Surgical procedure, *n* (%)		*P* value
	LLR group (*n* = 21)	LDG group (*n* = 42)	
Median age, years	68	67.5	0.84
Sex, male/female	13/8	23/19	0.59
Median body weight, kg	61	61.6	0.47
Median body mass index, kg/m^2^	23.8	23.6	0.79
Tumor location, upper/middle/lower	5/14/2	0/22/20	< 0.01
Preoperative distribution, normal/osteopenia/sarcopenia/osteosarcopenia	6/9/2/4	18/14/6/4	0.50
Median BMD	136.8	138.1	0.27
Median SMI	47.4	43.3	0.30
Median NLR	1.79	1.74	0.88
Median PNI	50.51	48.03	0.28
Median CAR	0.013	0.015	0.31
Median albumin level, g/dL	4.3	4.1	0.14

BMD, bone mineral density; CAR, C-reactive protein to albumin ratio; LDG, laparoscopic distal gastrectomy; LLR, laparoscopic local resection; m, mucosa; mp, muscularis propria; NLR; neutrophil to lymphocyte ratio; PNI, prognostic nutritional index; sm, submucosa; SMI, skeletal muscle index; ss, subserosa

### Relationship between surgical procedures and postoperative clinicopathological factors

The postoperative clinicopathological characteristics of the LLR and LDG groups are presented in Table 2. In the LLR group, all patients underwent sentinel lymphatic basin dissection, while in the LDG group, 1, 37, and 4 patients underwent D1, D1+, and D2 lymphadenectomy, respectively (*P* < 0.01). The sentinel lymphatic basins were classified into the following five regions: the left gastric artery basin (l-GA), right gastric artery basin, left gastroepiploic artery basin, right gastroepiploic artery basin, and posterior gastric artery basin [16]. Dissection of the l-GA was performed in 10 patients in the LLR group, whereas all patients in the LDG group underwent dissection in this region (*P* < 0.01). Both the resected specimen and pathological tumor sizes were significantly larger in the LDG group (*P* < 0.01). Postoperative complications of Clavien–Dindo grade ≥ 2 were observed in 13 (21%) patients, with a significantly higher incidence in the LDG group (*P* = 0.04). The most common complications included delayed gastric emptying (four cases, 6%) and anastomotic stricture (three cases, 5%). In the LLR group, postoperative complications occurred in only two patients, with one patient in the l-GA dissection group and one in the non-l-GA dissection group, showing no significant difference (*P* = 0.94). At 5 years postsurgery, body weight and BMI were higher in the LLR group; however, the differences were not statistically significant (*P* = 0.06 and *P* = 0.09, respectively). The distribution of normal, osteopenia, sarcopenia, and osteosarcopenia showed no significant differences between the two groups (*P* = 0.25).

**Table 2:** Relationship between surgical procedures and postoperative clinicopathological characteristics (*N* = 63)

Characteristic	Surgical procedure, *n* (%)		*P* value
	LLR group (*n* = 21)	LDG group (*n* = 42)	
Lymph node dissection, D1/D1 + /D2/sentinel node basin dissection	0/0/0/21	1/37/4/0	< 0.01
l-GA basin dissection, presence/absence	10/11	42/0	< 0.01
Median size of the resected specimen, cm	5	13	< 0.01
Median pathological tumor size, mm	13	24.5	< 0.01
Depth of tumor invasion, m/sm/mp/ss	9/1/11/0	23/0/18/1	0.29
Lymph node metastasis, pN0/pN1/pN2	19/2/0	34/6/2	0.49
Postoperative complications of Clavien–Dindo grade ≥ 2, presence/absence	2/19	13/29	0.04
Median body weight at 5 years postoperatively	58.4	51	0.06
Median body mass index at 5 years postoperatively	23.1	20.6	0.09
Postoperative 5-year distribution, normal/osteopenia/sarcopenia/osteosarcopenia	3/11/1/6	1/20/3/18	0.25

l-GA, left gastric artery; LDG, laparoscopic distal gastrectomy; LLR, laparoscopic local resection; m, mucosa; mp, muscularis propria; sm, submucosa; ss, subserosa

### Prognostic analysis determined by surgical procedures

**Fig. 2:**
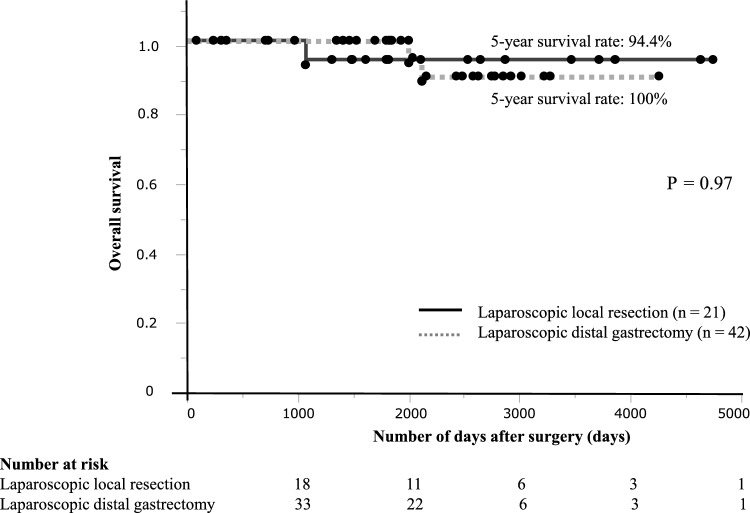
Kaplan–Meier curves for overall survival in patients who underwent laparoscopic local resection and laparoscopic distal gastrectomy

The 5-year overall survival was 94.4% and 100% in the LLR and LDG groups, respectively. No significant difference in overall survival was observed between the LLR and LDG groups (Fig. 2).

### Relationship between surgical procedures and SMI change rate

**Fig. 3:**
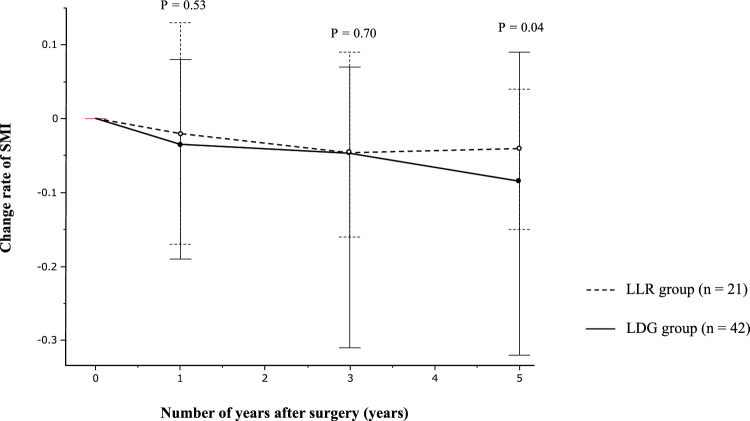
Comparison of the rate of change in the skeletal muscle index among groups who underwent laparoscopic local resection and laparoscopic distal gastrectomy. *LDG* laparoscopic distal gastrectomy, *LLR* laparoscopic local resection, *SMI* skeletal muscle index

When comparing the median change rates in the SMI at 1, 3, and 5 years between the LLR and LDG groups, the change rate from presurgery to 1-year postsurgery or from pre-surgery to 3 years postsurgery showed no significant difference in both groups. However, from presurgery to 5 years postsurgery, the LLR group showed a significant increase (Fig. 3).

### Relationship between surgical procedures and BMD change rate

**Fig. 4:**
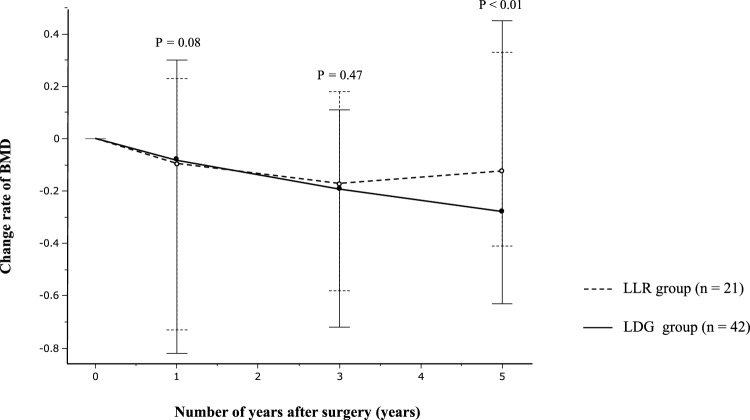
Comparison of the rate of change in bone mineral density among groups who underwent laparoscopic local resection and laparoscopic distal gastrectomy. *BMD* bone mineral density, *LDG* laparoscopic distal gastrectomy, *LLR* laparoscopic local resection

When comparing the median change rates in BMD at 1, 3, and 5 years between the LLR and LDG groups, no significant difference was found from presurgery to 1 year postsurgery or from presurgery to 3 years postsurgery in both groups. However, from presurgery to 5 years postsurgery, the LLR group showed a significant increase (Fig. 4).

### Relationship between surgical procedures and change rate of body weight and BMI

**Fig. 5:**
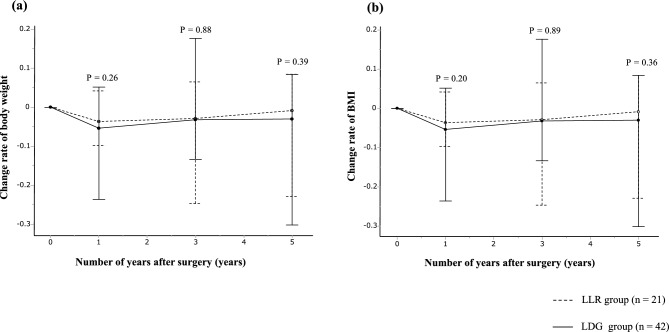
Comparison of the rate of change in body weight and body mass index among groups who underwent laparoscopic local resection and laparoscopic distal gastrectomy. (**a**) body weight; (**b**) body mass index. *BMI* body mass index, *LDG* laparoscopic distal gastrectomy, LLR laparoscopic local resection

Similar to the BMD and SMI change rates, the change rate of body weight and BMI was calculated. The median change rate of body weight and BMI at 1, 3, and 5 years postoperatively was compared between the LLR and LDG groups. Both groups showed a decrease from preoperative levels to 1 year postoperatively. However, from 1 to 3 years and 3 to 5 years postoperatively, the LLR group showed a greater increase than the LDG group, although the differences were not statistically significant (*P* = 0.39 and *P* = 0.36, respectively) (Fig. 5a, b).

### Relationship between l-GA dissection and the rate of change in SMI and BMD

**Fig. 6:**
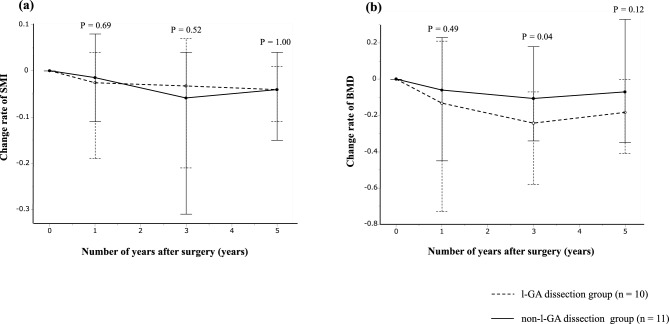
Comparison of the rate of change in skeletal muscle index and bone mineral density among groups who underwent l-GA dissection and non-l-GA dissection. (**a**) skeletal muscle index; (**b**) bone mineral density. *BMD* bone mineral density, *l-GA* left gastric artery basin, *SMI* skeletal muscle index

In the LLR group, patients were categorized into the l-GA dissection and non-l-GA dissection groups, and the median rates of change in SMI and BMD were calculated and compared for each group. In SMI, no significant differences were observed in its change rate from preoperative to 1 year postoperatively (*P* = 0.69), 1–3 years postoperatively (*P* = 0.52), or 3–5 years postoperatively (*P* = 1.00) (Fig. 6a).

In BMD, a significant difference was noted in its change rate from 1 to 3 years postoperatively (*P* = 0.04). However, no significant differences were observed in the change rate of BMD from preoperative to 1 year postoperatively or 3–5 years postoperatively (*P* = 0.49 and *P* = 0.12, respectively) (Fig. 6b).

## Discussion

In this study, we conducted a body composition analysis to evaluate the changes in the SMI and BMD following SNNS for EGC that was not suitable for endoscopic treatment. The study yielded the following results: (1) no significant difference was observed in overall survival between the SNNS and LDG groups; (2) patients who underwent SNNS showed an increase in the SMI at 5 years, postoperatively, whereas those who underwent LDG did not; (3) patients who underwent SNNS showed an increase in BMD at 5 years postoperatively, whereas those who underwent LDG did not; (4) the presence or absence of lymphadenectomy of the l-GA did not affect changes in SMI or BMD; (5) although no significant difference was observed, patients who underwent SNNS tended to have a greater increase in body weight and BMI than those who underwent LDG. To the best of our knowledge, this is the first study to suggest that function-preserving surgery for gastric cancer based on the sentinel node concept can prevent postoperative osteosarcopenia.

SNNS was developed to maintain quality of life after gastrectomy without compromising radicality or safety; surgical techniques and outcomes have been reported from various institutions [17, 18]. Kinami et al. reported that function-preserving surgery based on the sentinel node concept does not compromise oncological safety compared to standard surgery [19]. Similarly, the present study found no significant difference in overall survival between the two groups, indicating equivalent treatment outcomes.

The majority of patients with gastric cancer experience appetite loss, reduced oral intake, decreased digestive capacity, and body weight loss after surgery [20, 21]. Postoperative body weight loss, accompanied by reduced skeletal muscle mass, both lowers the effectiveness of postoperative treatment and correlates closely with decreased daily functional activities and quality of life, leading to a poor prognosis [22]. The results of the present study showed that even though both the SNNS and LDG groups experienced a reduction in skeletal muscle mass up to 3 years postsurgery, by 5 years postsurgery, the SNNS group showed an increase in skeletal muscle mass. This suggests that performing function-preserving surgery may be effective in preventing sarcopenia.

Postoperative osteoporosis after gastrectomy is an issue [23]. Maintaining bone density requires essential nutrients and proper exercise; however, after gastrectomy, nutrient malabsorption and reduced physical activity lead to bone loss [24, 25]. In patients with gastric cancer, osteoporosis reportedly increases the risk of death from the primary disease as well as from other conditions [26]. The findings of the present study indicated that, at 5 years postsurgery, patients who underwent SNNS showed an increase in bone density, suggesting that performing function-preserving surgery may offer the clinical benefit of preventing osteoporosis.

In gastric cancer surgery, retaining the residual gastric volume helps maintain nutrient absorption [27]. With SNNS, the residual gastric volume is preserved, which likely helps maintain nutrient absorption, gradually improving the SMI and BMD. Sarcopenia and osteoporosis have frequently been linked to postoperative prognosis in gastric cancer [28, 29]. We previously reported that osteosarcopenia, a condition in which both sarcopenia and osteoporosis are present, leads to worse prognosis compared to either condition alone in upper gastrointestinal cancer [13, 30]. Several studies have reported on lymphadenectomy of the l-GA and its association with delayed gastric emptying and body weight loss [31]; however, many aspects remain unclear. In this study, whether or not lymphadenectomy of the l-GA was performed in patients who underwent function-preserving gastrectomy based on LLR did not affect postoperative changes in skeletal muscle mass or bone mass. These results indicate that l-GA lymphadenectomy with vagal nerve branch dissection may not influence the development of postoperative osteoporosis or sarcopenia.

This study had several limitations. First, it was a retrospective study conducted at a single institution with a small sample size. Second, a bias was noted in tumor location between the LLR and LDG groups. These limitations could have introduced biases that might have influenced our results.

In conclusion, the findings of this study suggest that function-preserving surgery based on the concept of a sentinel node may contribute to preventing osteosarcopenia in patients with EGC.

## Data Availability

Research data that support the findings of this study are available from the corresponding author, TA, upon reasonable request.
